# The SINFONIA project repository for AI-based algorithms and health data

**DOI:** 10.3389/fpubh.2024.1448988

**Published:** 2024-10-23

**Authors:** Jorge Fernández-Fabeiro, Álvaro Carballido, Ángel M. Fernández-Fernández, Manoel R. Moldes, David Villar, Jose C. Mouriño

**Affiliations:** Galicia Supercomputing Center (CESGA), Santiago de Compostela, Galicia, Spain

**Keywords:** artificial intelligence-AI, cancer imaging, radiation risk appraisal, data repository, health data

## Abstract

The SINFONIA project’s main objective is to develop novel methodologies and tools that will provide a comprehensive risk appraisal for detrimental effects of radiation exposure on patients, workers, caretakers, and comforters, the public, and the environment during the management of patients suspected or diagnosed with lymphoma, brain tumors, and breast cancers. The project plan defines a series of key objectives to be achieved on the way to the main objective. One of these objectives is to develop and operate a repository to collect, pool, and share data from imaging and non-imaging examinations and radiation therapy sessions, histological results, and demographic information related to individual patients with lymphoma, brain tumors, and breast cancers. This paper presents the final version of that repository, a cloud-based platform for imaging and non-imaging data. It results from the implementation and integration of several software tools and programming frameworks under an evolutive architecture according to the project partners’ needs and the constraints of the General Data Protection Regulation. It provides, among other services, data uploading and downloading, data sharing, file decompression, data searching, DICOM previsualization, and an infrastructure for submitting and running Artificial Intelligence models.

## 1 Introduction

The technological development has entailed important changes in our way of life. It has transformed interpersonal communications, social networks, businesses, and education and it has also allowed us to deal with new challenges (1). Due to this progress, old and new inquiries have been tackled with new technological tools, and managing huge quantities of data has become a usual practice in the day-to-day in some scientific research fields as evidenced by the creation of large health data repositories such as The Cancer Imaging Archive (2) or the National Cancer Institute’s Imaging Data Commons (3), among others.

All that amount of data is expected to be stored to be requested when needed, so scientists have explored ways to increase access to information in the fastest and most feasible manner and began to create databases to store this information in an ordered way. The rise of high-speed connections through the Internet allowed to share and access data from anywhere in the world. The Human Genome Project (HGP) (4) was a big push for data repositories due to the amount of data generated. For instance, the U.S. National Center for Biotechnology Information hosted the data generated in the HGP in an already existing data repository known as GenBank (5), created in 1982 and whose size is now doubled every 2 years (6).

The technical developments have also driven research in medicine and health care. For instance, powered tools such as Computed Tomography (CT), Positron Emission Tomography (PET), or Magnetic Resonance Imaging (MRI), among many other available equipment, have improved the diagnosis of several types of cancer. The study of patients with cancer, as well as other pathologies, leads to a huge quantity of imaging and non-imaging data, that take up terabytes. Therefore, storing and managing them in an agile way represents a difficult challenge. Examples of how to deal with this situation are The Cancer Imaging Archive (TCIA) (2) and the National Cancer Institute’s Imaging Data Commons (3), whose purposes are identifying and hosting a large archive of cancer medical images and making them accessible for public download.

In this work, we present the data repository of the SINFONIA project (7). SINFONIA is an acronym taken out of “radiation riSk appraIsal for detrimeNtal eFfects from medical expOsure during maNagement of patIents with lymphomA or brain tumour,” a European Union (EU) project under the Horizon 2020 research and innovation funding program. The SINFONIA project aims to develop novel methodologies and tools that provide a comprehensive risk appraisal for detrimental effects of radiation exposure on patients, workers, carers, and comforters, the public, and the environment during the management of patients suspected or diagnosed with lymphoma, brain tumors, and breast cancers.

According to Allen et al. (8), lymphomas are the most common cancers in teenagers and young adults and the third most common group of cancers in childhood, after leukemia and brain tumors. Patient management includes an assortment of ionizing imaging modalities for staging and treatment selection. A lymphoma patient undergoes several low-dose but often full-body medical radiation exposures, in a wide variety of modalities, including those associated with treatment options (e.g., treatment planning CT, PET/CT imaging, Cone Beam CT [CBCT] for patient setup, etc.). Brain benign and malignant tumors are the most common solid tumors affecting children (9) and breast cancer accounts for 12.5% of all new annual cancer cases worldwide, making it the most common cancer in the world (10).

Detailed biokinetic, dose estimation and risk appraisal models require extensive data with significant storage needs and related computational burdens. Medical physicists and clinical researchers increasingly need an infrastructure that can provide the required computing capacity and capability for developing and testing novel models and services with potential assimilation in clinical practice. In addition, the increasing pervasion of machine learning algorithms in medical applications entails the integration of information repositories in research workflows. Anonymized patient images and relevant anonymized medical information can be shared among the research groups and can be used to test new Artificial Intelligence (AI) algorithms. In general, from segmentation masks on DICOM images (11) to imaging tumor detection (12, 13), AI has been demonstrated to be a powerful tool in health research.

The project plan defines a series of key objectives to be achieved on the way to the main objective, one of them to develop and operate a repository to collect, pool, and share data from imaging and non-imaging examinations and radiation therapy sessions, histological results and demographic information related to individual patients with lymphoma, brain tumors, and breast cancers. This repository aims to support SINFONIA researchers in managing various types of medical data, providing tools for imaging data visualization, as well as a platform for sharing and running inferences using AI models that have been developed and trained as part of their scientific activities within the project. This is strongly tied in with another of the key objectives of the project, which is to develop dose estimation tools based on personalized dosimetry methods, and advanced computational tools, powered by AI (14–21). As the process of training AI algorithms requires feeding large datasets into their models, the SINFONIA repository becomes a powerful tool for those researchers who want to share large amounts of imaging and non-imaging data for securely training them.

## 2 Materials and methods

### 2.1 The SINFONIA project

SINFONIA is a 4-year EU-funded project whose main objective of the project is to develop novel methodologies and tools that will provide a comprehensive risk appraisal for detrimental effects of radiation exposure on patients, workers, caretakers, and comforters, the public, and the environment during the management of patients suspected or diagnosed with lymphoma and brain tumors. A multidisciplinary consortium combining the expertise of 14 partners from eight countries (Austria, Belgium, Germany, Greece, Poland, Spain, Sweden, and Switzerland) work together to achieve this goal. The participants are major universities (OVGU, SU, UoC, UGENT, UJK, UNIGE), research centers (CESGA, EIBIR, NCBJ, SCK-CEN, SKANDION), hospitals (SCO, SERGAS) and industry partners (QAELUM). For clarity’s sake, the full names of the partners are unfolded in the nomenclature included in Section 9.

**Figure 1:**
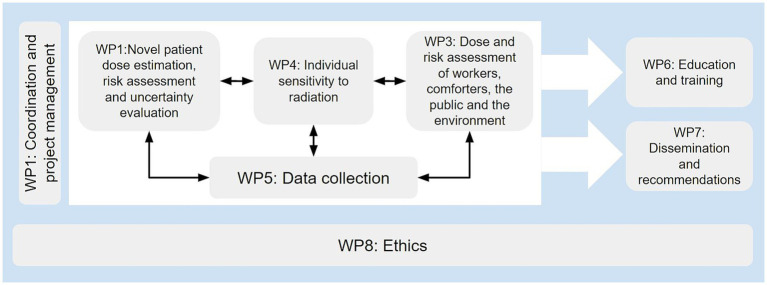
SINFONIA work packages.

The project workload is planned to be performed in 4-years running from September 2020 to August 2024 within eight work packages (WP), each of them having a leader institution. Work package 5 (WP5) is led by CESGA, as the technical expert in charge of the development of the repository presented in this paper. Since the repository is essentially a tool for partners to share their data and models, WP5 is directly related to WP2, 3, and 4 (the ones with the scientific workload of the project). Therefore, keeping a continuous interaction between CESGA as WP5 leader and the rest of the project members is crucial to keep the repository features and functioning aligned with their needs (Figure 1).

The number of organizations involved reflects the multidisciplinary expertise required for the achievement of all project tasks and goals to meet the expectations of participants, the scientific community, potential end-users, and the European Commission (EC). The organizational structure of SINFONIA is based on the best practices of international research projects, ensuring clear lines of responsibility and efficiency while enhancing collaboration and coherence. The consortium harbors extensive collective expertise and experience in the management and operation of EC-funded projects.

### 2.2 Repository development timeline and methodology

The project plan establishes for WP5 a set of tasks to be executed to implement a repository that meets the needs of the project: (i) definition of a platform architecture, based on functional (specific features that the repository should offer) and non-functional (how the repository should work in terms of performance, security, usability, etc.) requirements set by partners and considering the current regulations on data protection, particularly the General Data Protection Regulation (GDPR) (22) (Task 5.1), (ii) creation of the platform including data storage, computational capacities and protocols (Task 5.2), and (iii) integration of AI algorithms into the repository, where trained AI models would be tested, validated and deployed on the platform (Task 5.3). Moreover, as an EU-funded project, each WP must prepare a set of deliverables presenting their work for further EU validation (7). Regarding the repository, WP5 is expected to submit three deliverables: Deliverable 5.1 about the requirements and design of the prototype architecture (23), Deliverable 5.2 about the platform description, data model, and protocols (24), and Deliverable 5.3 describing the final platform and algorithms integration (25).

The definition of WP5 tasks required having a repository prototype up, and running, and ready to upload data at the end of the first year, and then releasing successive increments on an approximately yearly basis with both improvements and new features. This is aligned with a yearly prototyping software engineering methodology, which is a hybrid approach that combines two very common software development models: the rapid prototyping model, as defined by Crinnion (26), that “involves the creation of a working model of various parts of the software at a very early stage and after a relatively short investigation. That model becomes the starting point from which users can re-examine their expectations and clarify their requirements. When this goal has been achieved, the prototype model is thrown away, and the system is formally developed based on the identified requirements,” and the incremental model, as defined by Pressman (27), which “applies linear sequences in a staggered fashion as calendar time progresses […]. Each linear sequence produces deliverable ‘increments’ of the software […]. [It] delivers a series of releases, called increments, that provide progressively more functionality for the customer as each increment is delivered […].” As explained in Deliverable 5.1, the idea behind combining these two models is to organize the process with clear development increments in the form of usable versions, but at the same time allow a continuous and iterative flow of information about eventual issues and proposal of new or improved features.

**Figure 2:**
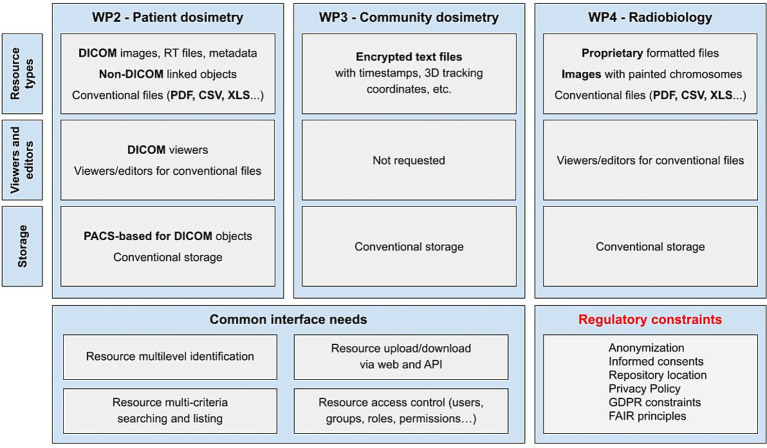
Work packages’ analysis of needs.

The development process started with the collection of requirements from project partners through a set of *ad hoc* questionnaires composed within WP5, validated by the Executive Board of the project, and then circulated among the partners during the first months of the project. These questionnaires, which can be found in full in Deliverable 5.1, survey the partners about the type and amount of data expected to be uploaded to the repository, the features they would like to find the repository, and a first approximation to the AI algorithms expected to be uploaded to the repository. Figure 2 summarizes the needs identified from those questionnaires along with other external constraints such as data protection regulations. These needs are mainly those of WP2, WP3, and WP4 as these are the ones that would share health data and trained AI models through the repository.

In summary, partners expected the SINFONIA repository to allow them to upload and download data; search for files; store, manage, and view different types of files, with a special interest in the DICOM format; and to have at least basic privacy measures such as access control lists for setting different permissions levels for their data resources. Given its relevance in the scientific field in which the SINFONIA project is involved, we will pause for a moment to present the DICOM standard. DICOM, the acronym for Digital Imaging and Communications in Medicine (28), is the international standard (ISO 12052) for medical images and related information. It defines the formats for medical images that can be exchanged with the data and quality required for clinical use. It is implemented in radiology, cardiology, and radiotherapy devices, and it is increasing its presence in medical devices from other domains such as ophthalmology or dentistry. In addition to DICOM images, an amount of non-imaging data is generated during the management of an oncological patient. That is why the SINFONIA repository also allows to storage and share any kind of file like other cloud-based file management systems do.

Having all these needs identified, an analysis of available technologies was carried out through a survey of websites, source code repositories, and computer science bibliography, to lay the foundations for the development of the repository. The analysis is summarized here, but the detailed survey is available in Deliverable 5.1.

Provided that the SINFONIA repository is essentially a tool for sharing and storing health data, the available medical image collections and repositories were reviewed first. The search was based on the collection of repositories gathered in Open-Access Medical Image Repositories (29), CVonline Image Databases – Bio/Med (30), and Neuroimaging Tools & Resources Collaboratory – Registry (NITRC-R) (31) websites. Some of them are devoted to storing medical imaging data, whereas others provide support for the whole lifecycle of data, from experiment designs to the publication of results. Some cover a more general scope and are designed for non-specific fields, and others are tailored to disciplines like neuroimaging.

After this review of the main advantages and drawbacks found on the different tools reviewed, we found in Girder (32) the middle-ground option to be the basis of the SINFONIA repository. It is a free and open-source platform meant to enable the quick and easy construction of web applications that need to manage data that are dynamically provided by users of the system or exposed through external data services. The features it provides are very close to those of file clouds, which allows users to store files of any type and to freely organize them in custom folder hierarchies. The fact that Girder incorporated a plugin to preview both the images and the metadata of the DICOM files was another important point in favor of its choice as the basis for the prototype.

The development of the first-year prototype followed the mentioned rapid prototyping model and consisted of the deployment of a Girder instance with some customizations proposed by users after some validation rounds. That prototype was released and officially presented to partners in the consortium meeting held at the end of the first project year.

**Figure 3:**
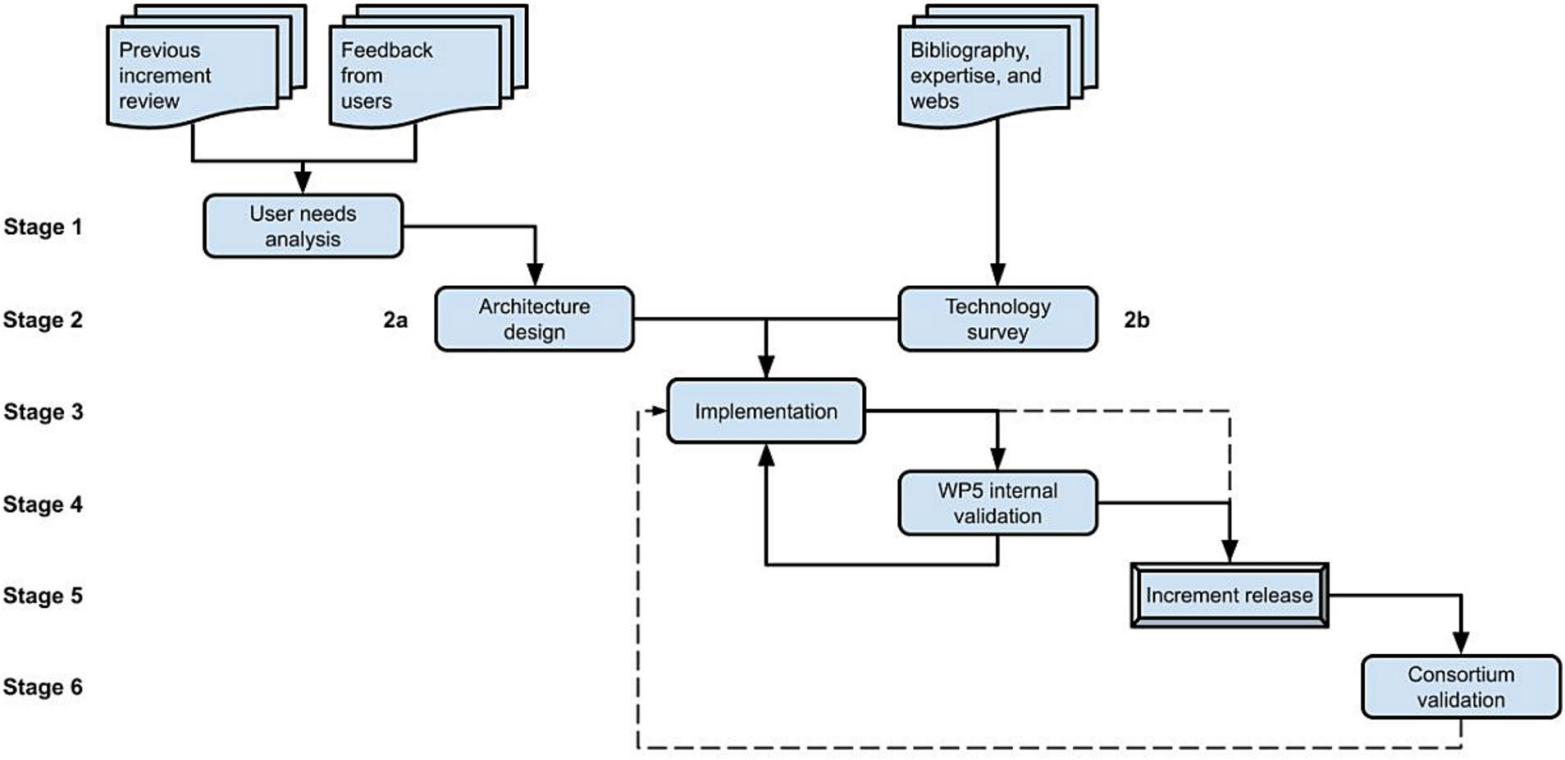
SINFONIA repository’s yearly prototyping methodology from second year onwards.

The development of versions from year 2 onwards followed the yearly prototyping outlined in Figure 3. Based on the previous version and the feedback received from users, we review whether their needs have changed, or new ones have arisen (stage 1), refine the design of the architecture if necessary (stage 2a) and proceed with the implementation (stage 3) of improvements (stage 3), new functionalities and bugfixes, reviewing and expanding the information collected about the available technologies (stage 2b) if the complexity of the work to be done requires it. The loop between implementation and internal validation (stage 4) is supported by keeping two simultaneous versions of the repository coexisted (Pre-production and Production), which is a common practice in software development. The Pre-production serves as the internal WP5 sandbox for testing and validating improvements, new implementations, and bugfixes, being released as a Production version for all project members (stage 5) approximately yearly (before the consortium meetings), but also when major novelties or bugfixes merit it, which led to some intermediate extra releases between the planned ones. After each release users are encouraged to make their tests and contact the development team back with suggestions or bug reports (stage 6). Depending on the complexity of the implementation of the received feedback and the severity of the shortcomings or bugs detected (if any), changes are made on the fly (loop back to stage 3) or just considered for the next major release (input for stage 1). Table 1 summarizes the history of versions of the repository year by year.

**Table 1:** History of repository’s main milestones.

Description	Year + Quarter
Project members’ analysis of needs and review of anonymization procedures and GDPR	Y1Q1
First architecture definition, extensible for further iterations	Y1Q2
Implementation of the prototype repository	Y1Q3
Internal validation of prototype and public release	Y1Q4
Architecture refinement to fulfil project’s requirements and project member’s needs	Y2Q1
Implementation of year-two-version of the repository	Y2Q3
Internal validation and year-two-version public release	Y2Q4
Definitive architecture design supporting execution of AI models	Y3Q1
Implementations of year-three-version of the repository	Y3Q4
Internal validation and public release of new version with first AI algorithms execution platform	Y4Q2
Implementation of final version of the repository	Y4Q3
Internal validation and public release	Y4Q4

### 2.3 Repository’s architecture

The contents of this subsection summarize the parts regarding the repository’s architecture from the first two WP5 deliverables of the SINFONIA project (23, 24), so any further information can be found in these public documents.

#### 2.3.1 Overview of the architecture

**Figure 4:**
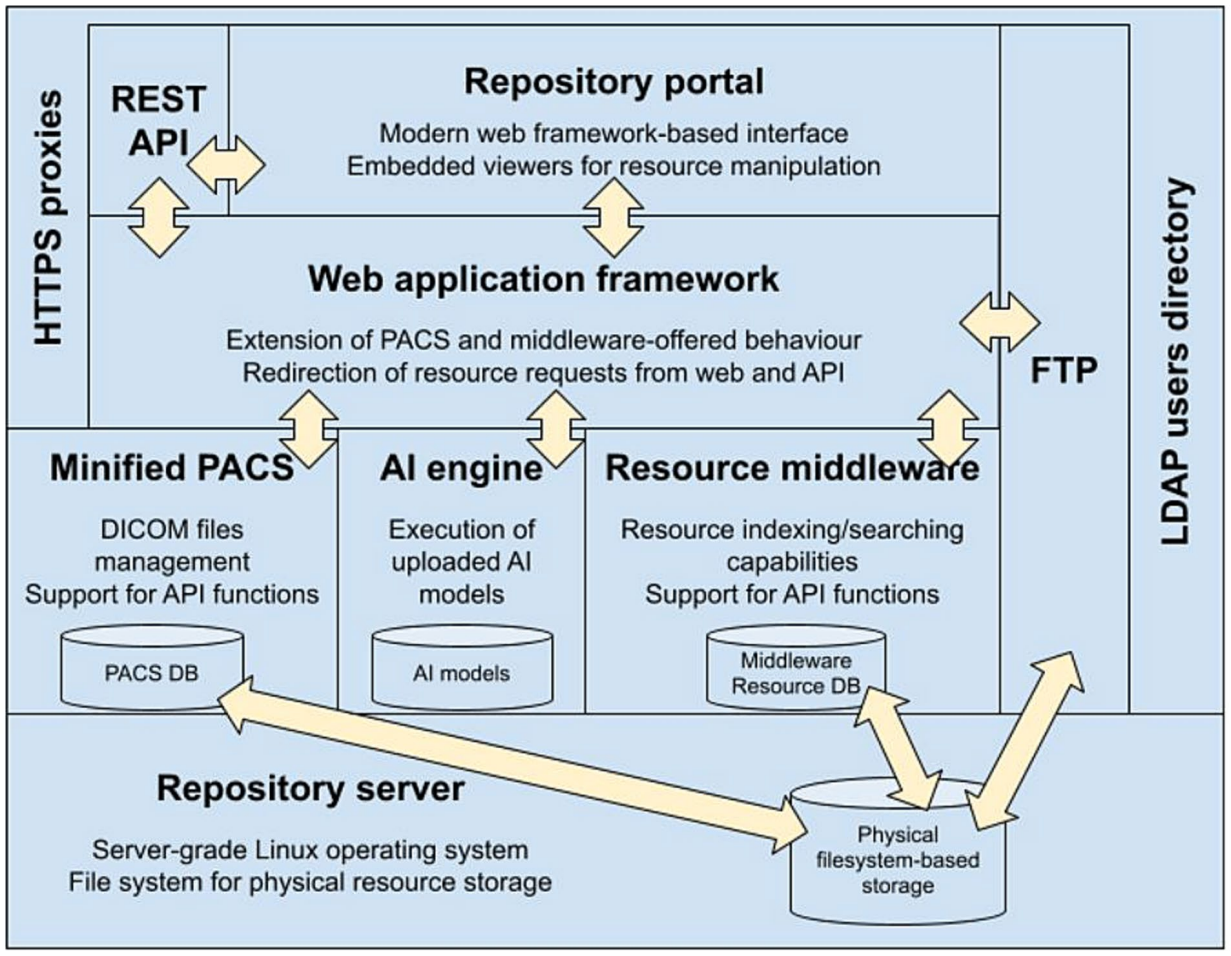
Block diagram of the final platform architecture.

According to Carnegie Mellon University’s Software Engineering Institute, the software architecture of a system represents the design decisions related to overall system structure and behavior (33). Architecture helps stakeholders to understand and analyze how the system will achieve essential qualities such as modifiability, availability, and security. Figure 4 summarizes the architecture of the SINFONIA repository we are going to discuss next.

This architecture fits into the 3-tiered model (34), a software architecture pattern widely used in web applications composed of three layers: presentation (the portal and the application programming interface, or API), logic [split between the middleware and the Picture Archiving and Communication Systems (PACS) (35)], and data (distributed among the databases that index and store the managed resources). These three layers rely on a repository server on which their codebases are run, and data is physically stored.

From the point of view of the tasks performed by each tier of the architecture, it fits in a hierarchical Model-Viewer-Controller (MVC) architecture pattern (36), since the internals of part of the components are following by themselves a full MVC pattern. The central component of this pattern is the Model, which represents the information structure of the application. A View is any representation of information, which in web applications are usually HTML templates that are displayed in the user’s browser filled with the specific data retrieved. The Controller just accepts input and converts it to commands for the model or view.

In our case, the Model part is the schema and the contents of the middleware database, the Controller part is the middleware application code that runs on the server, and the View part is the web portal pages through which users interact with the repository. The minified PACS, the Resource Management Middleware, and the AI engine (these three components are explained later in this subsection) will offer their respective programming interfaces (View) through which the web framework will be able to make the necessary requests. Said requests will be serviced by their respective application codes (Controller) that will query and/or make the necessary changes in the corresponding databases (Model).

The entry point for users into the repository will be either the RESTful API (that is, an interface that allows other programs, tools, or services to interact with it) or the Repository Portal, both communicating with each other as all Django views interact through the API. Both the RESTful API and the Repository Portal are supported by a Web Application Framework able to build and serve web pages and process user requests. This Framework serves the web portal and the RESTful API by itself, allowing us to extend or adapt its behavior according to further evolutions of the architecture. REST is an acronym for “Representational State Transfer,” that means that resources traded in a web application (data or objects) are represented by specific URLs (uniform resource locators). When a client requests a resource, the server sends a representation of that resource. The state of the resource is transferred between the client and server through these representations, allowing the client to interact with and modify the resource without needing to store information about previous interactions.

The Resource Management Middleware plays the roles of Model and Controller, that is, it relies on the support for indexing and storing non-DICOM resources, and for managing users, groups, and permissions that SINFONIA users expect to manage through the repository. At the same level, there is another component called “Minified PACS,” introduced to provide enriched support of the so-called “DICOM model of the Real World,” see Section 3.3.2. Minified PACS is a lightweight PACS for research purposes, as opposed to full PACS (37), built for digital images that must be efficiently stored, rapidly retrieved, captured from multiple acquisition modalities, and simultaneously accessed from multiple sites in clinical environments.

The AI engine powers the repository AI execution platform. It stores the models submitted by users and integrates the software required for their execution, mainly Python packages for models based on the most common machine learning frameworks (Tensorflow and Keras) as well as an instance of the MATLAB Compiler Runtime for models implemented with this technology. Those models are exposed through an internal API that allows the upper layers to send requests to get information about them (descriptions, inputs, outputs) and to run them.

The core of the architecture is complemented by two other components to provide two additional services. First, a Secure File Transfer Protocol (SFTP) (38) enables massive data transfers that could be accessible from the Web Framework to allow users to move resources between the repository and the user’s local computer. Second, a Lightweight Directory Access Protocol (LDAP), an open, vendor-neutral, industry-standard application protocol for accessing and maintaining distributed directory information services over a network (39). LDAP provides centralized storage for all users’ credentials so that different applications and services can connect to the LDAP server to validate users’ requests on authenticated resources (40).

Finally, all the repository infrastructure would rely on a server connected to either a local or a network-attached physical storage on which the data resources indexed by the middleware are going to be effectively kept.

#### 2.3.2 Platform implementation

Let us proceed to the implementation details of the architecture. Several software tools were used, each of them with its particularities and not essentially thought to work together. The successful integration of all these components is crucial to achieving a final version of the repository that complies with the expected functionalities.

**Figure 5:**
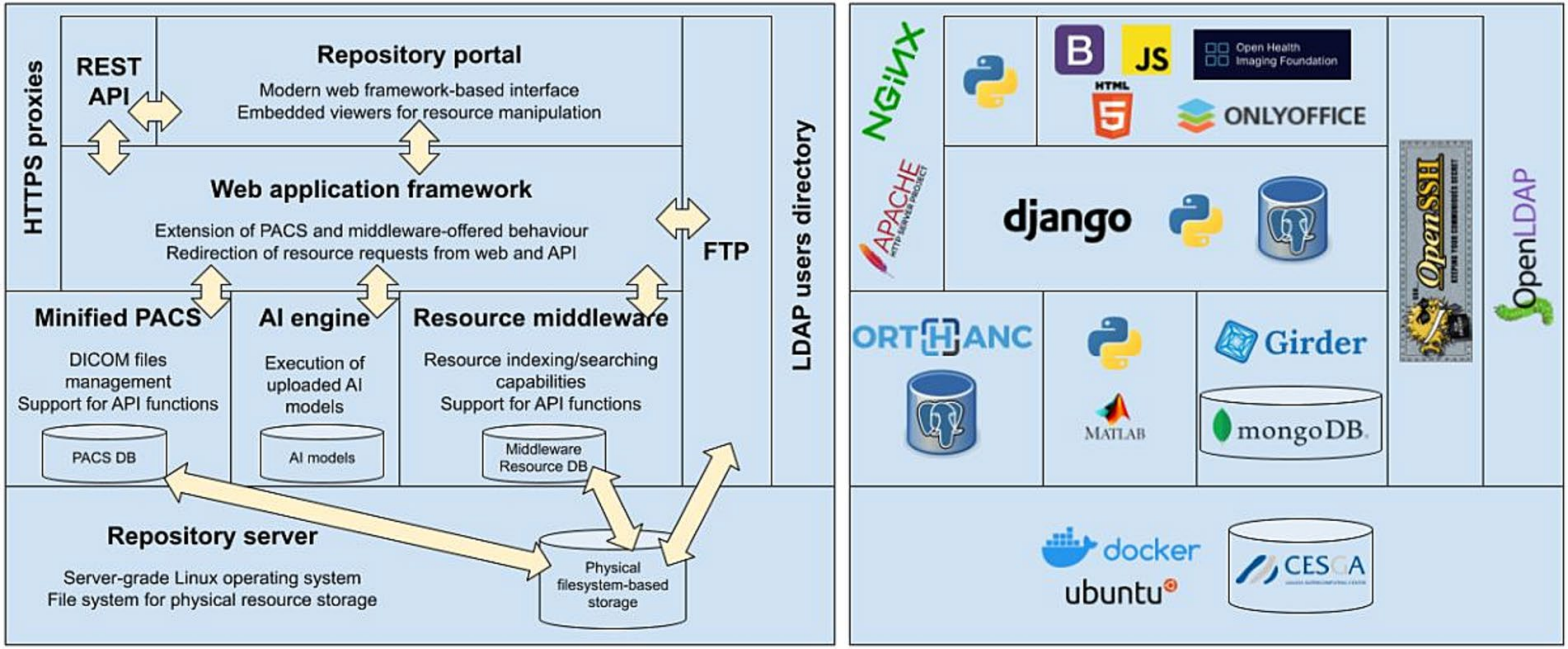
Correspondence between implemented software and platform architecture.

Figure 5 shows the correspondence between the different components of the architecture described in the previous section and the software tools and programming frameworks used to implement and interconnect those components.

**Figure 6:**
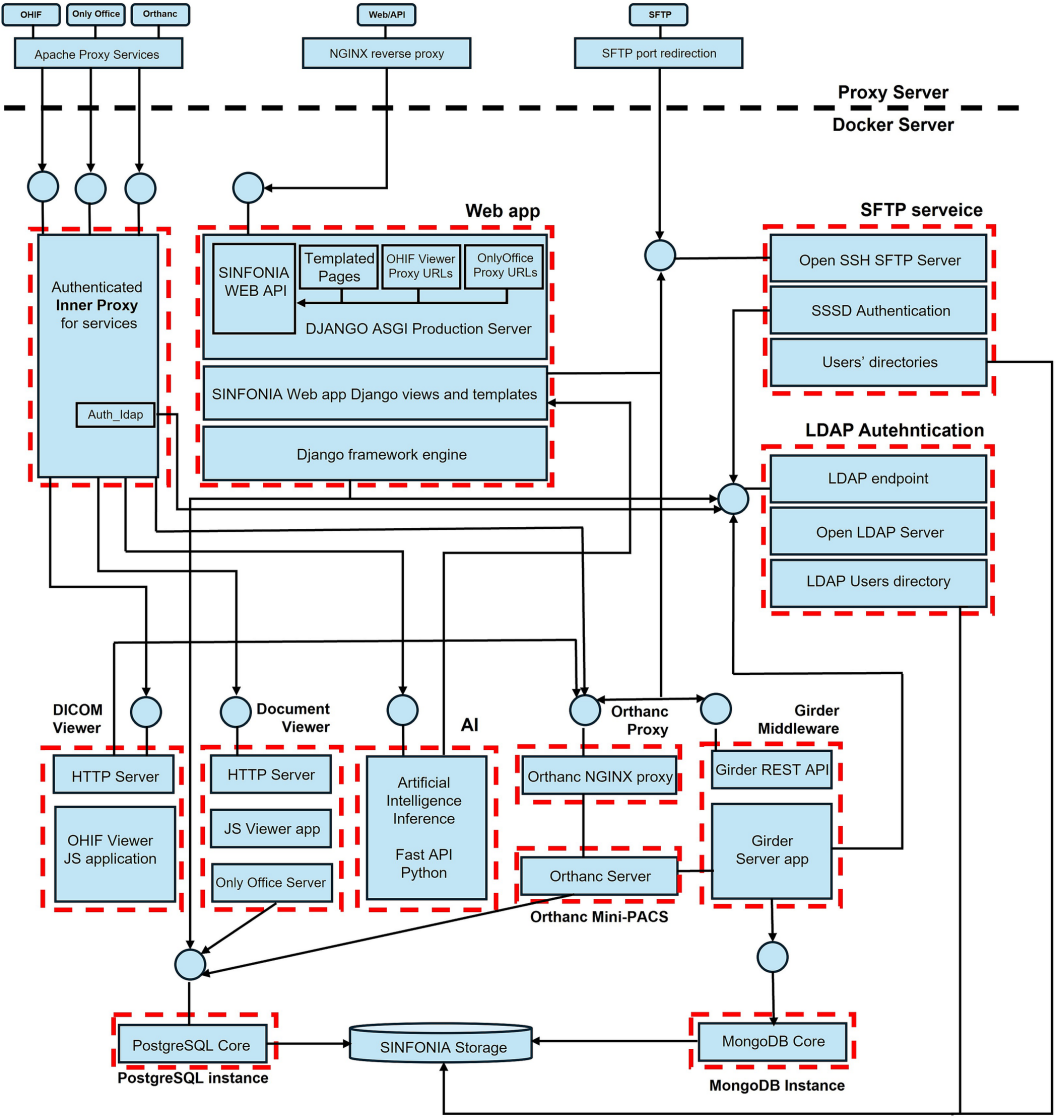
Unfolded diagram of the repository architecture implementation.

Most of the components of the SINFONIA platform are assembled as Docker images and deployed as containers (41). Figure 6 elaborates on the representation of components and tools from Figure 5 by revealing their internal software stacks and outlining explicitly the dependencies between them. The boxes with red dashed lines represent the Docker containers that make up the platform implementation, with the circles that receive arrows representing the port-based endpoints of those containers.

A customized Girder is part of the core of the platform, playing the role of Resource Management Middleware, that is, the underlying instance of a software solution able to index (usually through a database), store, and serve the different types of files and metadata that SINFONIA users expect to manage through the repository. Girder was selected because, after some customizations, it allowed us to quickly deploy the first version of the repository. Moreover, Girder also provided a previewer of DICOM images together with its metadata that fit the aims of the repository. In the final version, Girder was relegated to the backend.

By design, Girder relies on a MongoDB database (42) on which the resources uploaded to the repository are indexed within Collections, Folders, and Filesets, along with information regarding Users, Groups, and Permissions. The customization relies on some additional modifications to improve the way the DICOM plugin indexes the files in MongoDB, making it aware that there is an Orthanc instance effectively managing and storing them.

**Figure 7:**
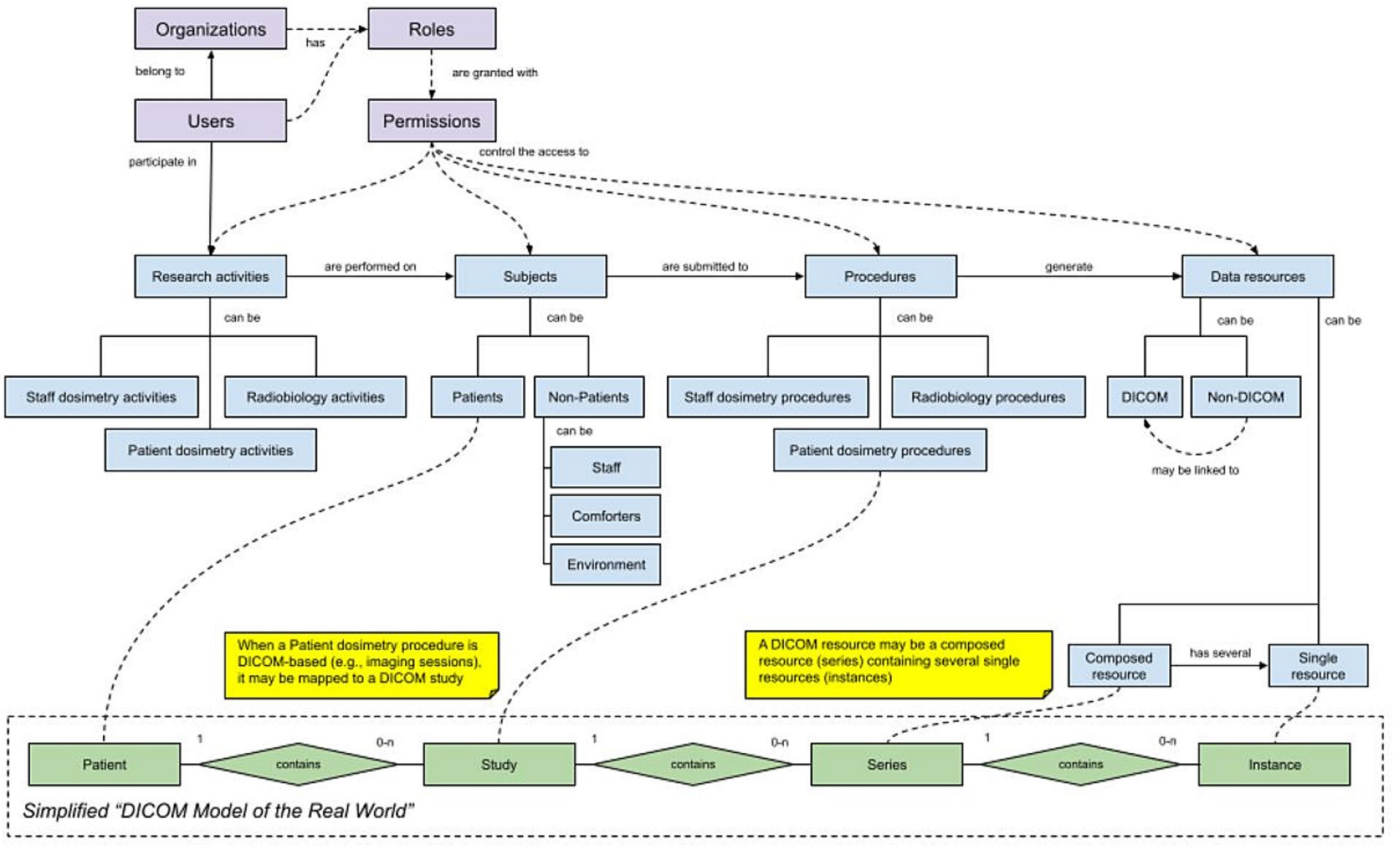
SINFONIA data workflow and DICOM model of the real world.

Orthanc (43) is an open-source DICOM server that condenses the functionalities of a PACS into a lightweight implementation (i.e., a “Minified PACS,” as referred in the architecture overview) that is not subject to the strict medical-device requirements and certifications of other equivalent systems intended for clinical practice in healthcare centers. This makes Orthanc especially useful for research environments such as the SINFONIA project. Orthanc implements the DICOM standard and its “Model of the Real World,” which represents DICOM resources as coming from patients that can be submitted to several studies, with each study containing several series, and each series being a collection of DICOM files named instances, so it is well suited to better support that part of the information model (see Figure 7).

Orthanc can store the DICOM files directly in an internal folder on the underlying filesystem or integrate them into the same database it keeps for indexing them and for managing other ancillary information such as the metadata, the history of changes, and a map of a selected number of DICOM tags per file to avoid repeated accesses to the storage. It also implements the DICOMweb standard (44), thereby offering services to retrieve, store, and search for DICOM resources through a RESTful API. This enables the option to connect external components that expect DICOMweb-based endpoints, such as a DICOM web viewer able to supersede the Girder-based previewer.

Regarding the Web Application Framework, we opted to use Django, a free and open-source, Python-based web framework with comprehensive documentation and a large user base that allows developers to take web applications from concept to launch in a very short time (45). Django follows a Model-Template-View (MTV) architecture which implies slight internal differences concerning the separation between the layers of a pure MVC architecture as the one we implemented. However, at this level of review, we can consider that it practically follows the latter book of Holovaty et al. (46). In the SINFONIA web application, the role of the MVC Controller is played by the Django views, which build and send requests to the Girder and Orthanc APIs, which may respond with both JSON objects (information to be displayed, in general) and binary objects (files to be downloaded or previewed, for example).

As said, one of the features of the SINFONIA repository is the implementation of an external DICOM viewer. The chosen one was OHIF (47), an open source, web-based, medical imaging platform which provides a core framework for building complex imaging applications to support imaging research (47). It supports DICOMWeb as an image source, so that it can be used to display DICOM files managed by Orthanc, and also provides tools used day to day in health research and clinics such as measurement and image annotation, 3D segmentation, customized display layouts for efficient interpretation of medical images, etc.

The need to deal with non-imaging data such as medical reports, spreadsheets, etc., made us include the web-based OnlyOffice (48) document viewer, which is easily enabled by providing the JavaScript code of the OnlyOffice viewer component with a valid URL to download the file to review. For that purpose, a Django view reacting to such requests and sending them down to the Girder middleware server has been implemented. This document viewer is compatible with popular formats from both commercial (Microsoft Office’s OOXML and Legacy formats) and open-source office suites (LibreOffice’s OpenDocument formats), as well as with other commonly used files such as PDFs, plain text files, HTML pages, EPUB electronic books or CSV files. See more information in the Deliverable 5.2 (24).

One of the specific tasks the SINFONIA repository had to achieve was the implementation of an AI area where the researchers could share their trained AI models and run them inside the repository, even using data hosted in the repository as inputs. The AI area is built in its own Docker container where Python is installed, together with some of the most common AI packages Python provides: Tensorflow, Keras, etc. Moreover, a Dynamic Package Installation was implemented to automate the installation of the packages a submitted model requires. For MATLAB-compiled AI models, the MATLAB Runtime (49) was installed in the AI engine container. The installed versions are the specific ones used by the project partners that produce MATLAB models to compile them.

**Figure 8:**
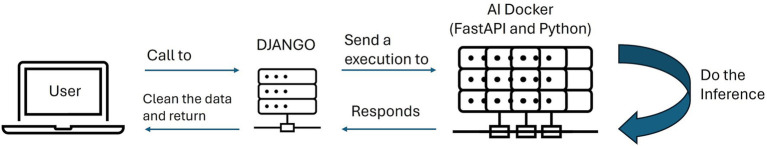
AI inference workflow.

Users interact with the AI engine through the Web Application Framework, Django, for both model submission and inference running requests. See Figure 8 for a diagram of the inference workflow. The implementation of the submitted models is not automatic for two main reasons. On the one hand, the amount of different frameworks and programming languages an AI model could be trained with could make the storage cost of building such a Docker container unaffordable. On the other hand, allowing the automatic execution of programs inside the repository would be a security hole we opted to avoid.

Regarding the data hosted in the repository, it is managed by both Girder and Orthanc, each of them having its underlying database to index the data. Girder uses MongoDB, which is a NoSQL database system that gathers data in collections of Binary JSON (BSON) (50) objects. Orthanc’s underlying database is deployed on a shared PostgreSQL (51) instance, on which the office document viewer also keeps an internal database. PostgreSQL is an open-source object-relational DBMS (Database Management System) whose origins date back to 1986 as part of a DARPA-sponsored project, having a core platform with more than three decades of active development.

**Figure 9:**
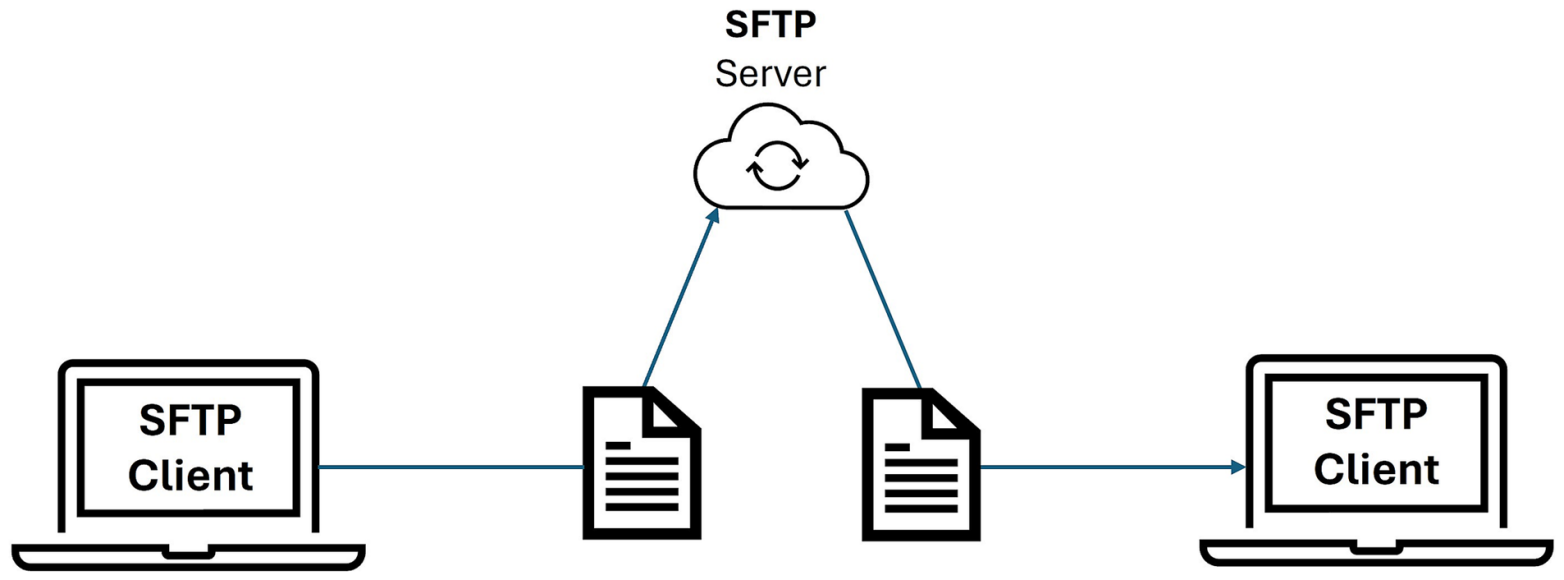
SFTP workflow.

The amount of data expected to be managed with the repository encouraged the implementation of an SFTP server for massive secure file transfer over a network (see Figure 9). The main advantages of SFTP are the protection of data in transit through encryption, its speed for transferring large and/or multiple files simultaneously, its seamless integration with Virtual Private Networks (VPN) and firewalls, and the possibility of accessing servers through friendly interfaces such as web portals or SFTP Graphic User Interface (GUI)-based clients. In our case, we have configured an OpenSSH-based (38) SFTP server that sets a separate directory tree for each user of the repository. This way any attempt by a user to access files or directories outside of their tree will fail, as they do not have the permissions to access anything beyond their own space in the SFTP server.

All the authorization and authentication operations within the repository rely on an instance of an OpenLDAP (52) directory, an open-source implementation of the LDAP protocol. All repository services that require prior authentication check the validity of the credentials provided with an OpenLDAP server deployed on the platform.

**Figure 10:**
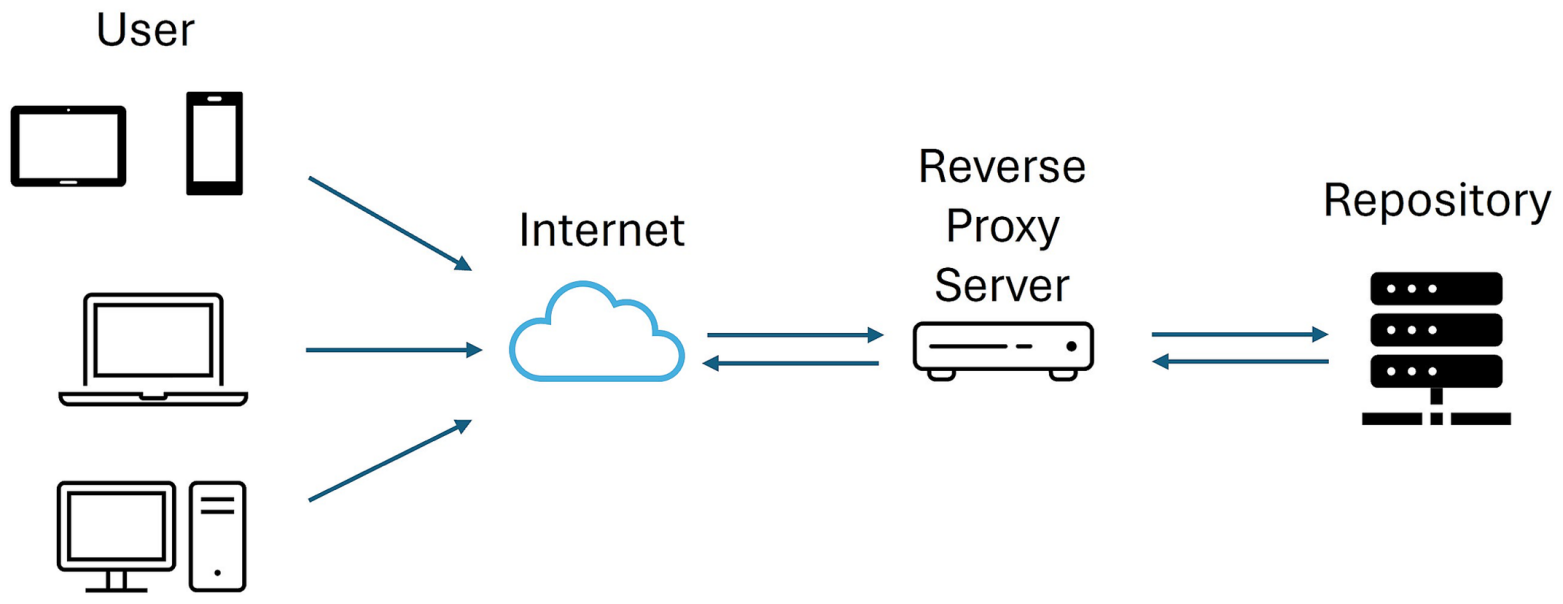
Reverse Proxy Server Workflow.

Three components live in the so-called “Proxy server” machine, in contrast to all other components, which are Docker containers (outlined with red dashed lines) running inside the “Docker server” machine. The first of these three components is the NGINX (53) reverse proxy, deployed to act as a reverse proxy server, that is, a server put in front of another one to retrieve resources or attend to requests on behalf of the hidden one (see Figure 10). The second one is the “Apache external proxy” (54), which captures and redirects the public requests of external services such as the OHIF DICOM or the office documents viewer. The third non-containerized component, “SFTP service endpoint,” is just a redirection from a public port pair in the proxy machine to the SFTP port exposed by the Docker container.

In general, proxy servers improve the security of Internet-exposed infrastructures by (i) encrypting the connections under the HTTPS protocol, (ii) dismissing requests from allegedly malicious IP addresses previously included in an access control list, and (iii) avoiding the direct public exposure of their components. In particular, the separation of the proxy server into two different instances, each running a different tool, is simply because the integration of some of the underlying services was more convenient with NGINX and others with Apache.

### 2.4 Technical validation

The software engineering process combined the generation of public yearly versions to be validated by the whole project consortium with a continuous internal revision within WP5. So, once a relevant increment (new or improved features, major bug fixes, etc.) of the repository was internally tested by CESGA, it was shared with the rest of WP5. Those members conducted their tests, sending feedback about dysfunctionalities, bugs, and possible improvements. When this internal validation was achieved, a yearly platform was presented to the SINFONIA consortium, which validated it and proposed new features they expected the repository to have in the future.

### 2.5 Regulatory constraints and ethics

According to Deliverable 5.1 (23) of the project, one of the specific challenges that SINFONIA pledged to address was to take into consideration the risk communication and the ethics of medical applications. To achieve this, the SINFONIA proposal states that all data managed in the repository must be stored securely and de-identified to remove all patient identity-related information. The main origin for these constraints is GDPR, Regulation 2016/6797 of the European Parliament and of the Council of 27 April 2016 (22). The territorial scope of this regulation includes all member states and it must be fulfilled by any European corporation, regardless of its headquarters being allocated in the rest of the world.

The SINFONIA repository and its underlying data storage are deployed in CESGA’s computing and storage infrastructure, that are physically hosted in its facilities in Santiago de Compostela (Galicia, Spain). Consequently, the repository must comply with the Spanish Organic Law on the Protection of Personal Data and Guarantee of Digital Rights (LOPD-GDD, from its Spanish official denomination “Ley Orgánica de Protección de Datos Personales y garantía de los derechos digitales”) (55), which implements the GDPR. Both GDPR and LOPD-GDD share the aim of ensuring the free flow of personal data between member states. All partners of the SINFONIA project are aware of how sensitive the data they are managing is. We have identified three main regulation-derived constraints that must be integrated into the development of the SINFONIA repository. Firstly, the data collected, generated, and/or related to the SINFONIA project should not be processed out of the GDPR territorial scope when interacting throughout the repository. Secondly, data navigation, uploading and downloading must be somehow restricted in those cases in which data owners are mandated to limit access to their resources. Such control gives rise to an additional requirement as it implies the creation of an account-based authentication and authorization system that collects some basic users’ personal information that must be processed according to a GDPR-compliant Privacy Policy. Thirdly, regarding the assurance of the proper de-identification of the data resources managed by the repository, although GDPR obliges the repository to ensure the de-identification or removal of personally identifiable information, it is the users’ responsibility rather than the repository administrators. That is, the de-identification must be performed on the site of origin before uploading the data to any server.

The SINFONIA partners agree that imaging and non-imaging files in different electronic formats contain metadata that could include personal information, so before uploading any file to the repository, that metadata must be de-identified. It is in this sense that some basic technical guidelines about the de-identification of files and images have been prepared to help the partners minimize the chance of any personal information leak while sharing data through the repository (56).

The importance of electronic health data and the urgency to make progress toward a common policy in response to health emergencies has increased since the COVID-19 pandemic. In this direction, the European Union has proposed the establishment of domain-specific common European Health Data Space (EHDS). The establishment of this common European data space is developed in the proposal for a Regulation of the European Parliament and of the Council on the European Health Data Space 2022/0140 (57).

The EHDS aims to increase the sharing of health data for primary and secondary uses. The former is the provision of health care to maintain or restore the state of health of the patient, including the prescription and provision of medicinal products, while the latter refers to the classic notion of scientific research and the innovation of new products or services related to public health or social security.

The SINFONIA repository contains patient data precisely for secondary use, and both the regulation and the new proposal reinforce the privacy of natural persons. So, their health data should be made available in a pseudonymized format and data users should not attempt to re-identify natural persons from datasets. All those new constraints are met with by the data held in the repository as it must be previously de-identified regarding the GDPR constraints as mentioned above.

## 3 Results

The SINFONIA repository was designed to be a cloud-based environment that provides services such as data uploading/downloading, data sharing, file decompression, data searching, DICOM previsualization, and an infrastructure for submitting and running AI models. This paper just introduces the main characteristics of the repository, being further details thoroughly described in the project’s deliverables. Regarding the yearly prototyping methodology, several versions of the repository existed during the project timeline. However, although these intermediate versions were important to build the repository, they are no longer available. Then, we only describe the final version of the repository and not a history of all versions, whose main milestones are summarized in a quarterly basis in Table 1.

At the time of writing this paper, the SINFONIA repository is a completely functional data repository with all the features discussed here, including the AI execution platform. It is deployed as a dedicated project within the CESGA’s OpenStack cloud infrastructure. The container server is a virtual machine (VM) with eight virtual CPUs, 32 GB of virtual RAM, and a virtual hard drive of 60 GB for internal use (project data is not kept there but in a 1 TB dedicated zone of CESGA’s storage infrastructure). The reader can refer to the first two deliverables (23, 24) for more technical details.

**Figure 11:**
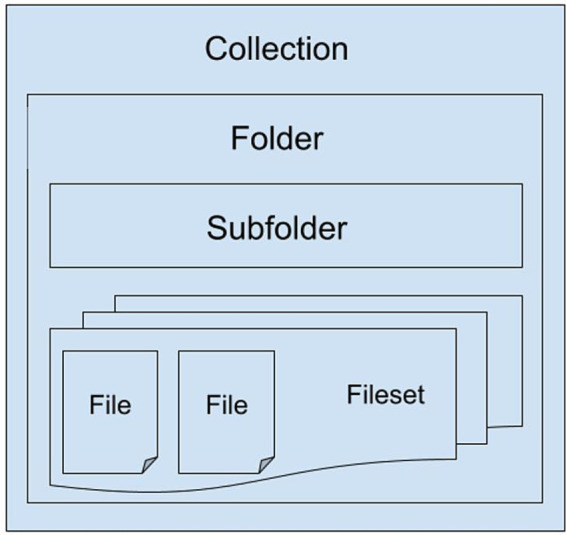
SINFONIA repository folders’ hierarchy.

Registered users can store files of any kind in the repository and share them at their will with other registered researchers or groups within the project. Data hierarchy is inherited from Girder, and it is organized, at the top level, in separate collections. Inside a collection, data can be organized in a tree-like structure of folders, and folders can contain subfolders or filesets, the latter ones being collections of several individual files (see Figure 11). Access to collections and folders are individually managed though access control lists, that allow their owners to make them private or public and to decide which of the other users can read, modify or manage them.

**Figure 12:**
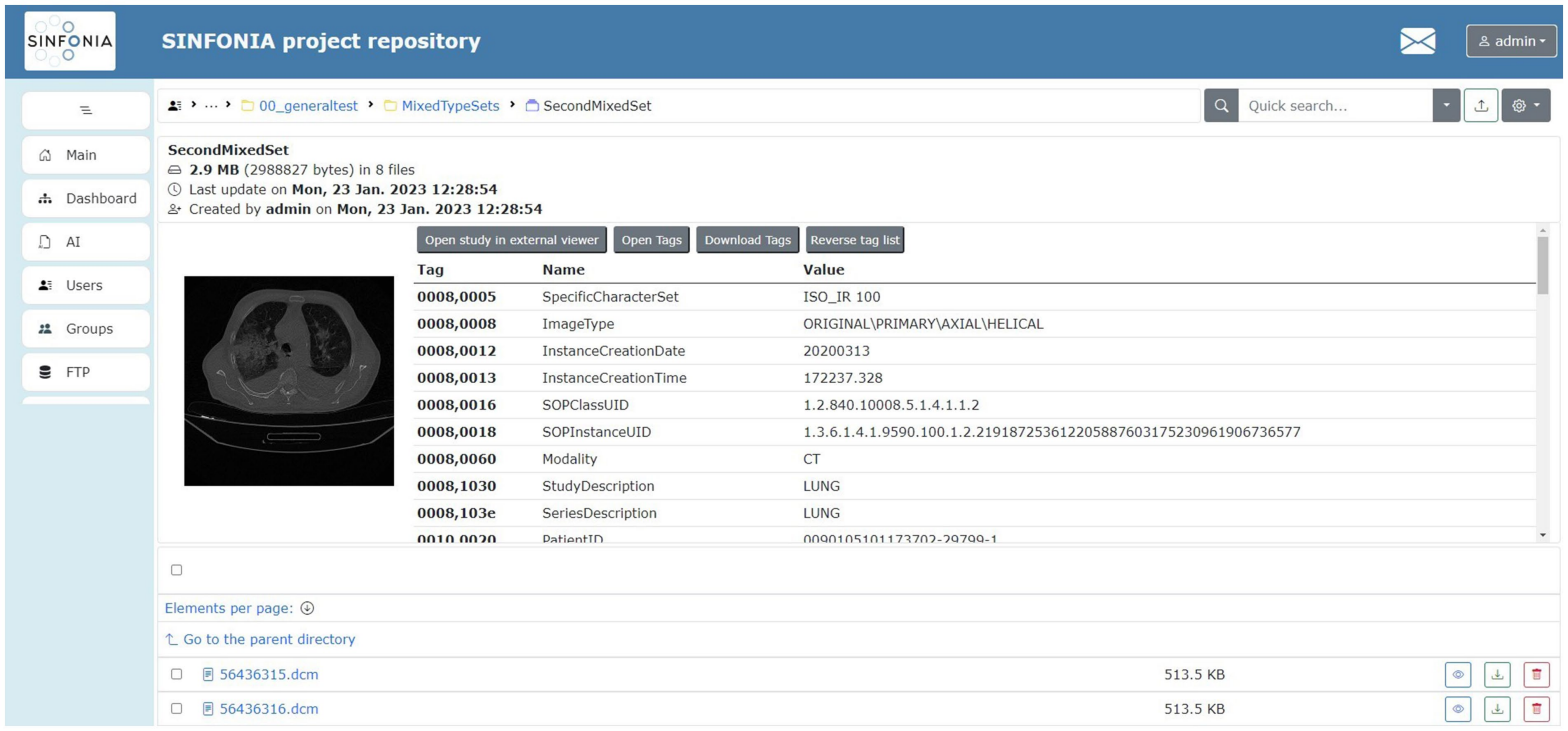
SINFONIA repository DICOM previewer. Adapted with permission from “COVID19-CT-Dataset: An Open-Access Chest CT Image Repository of 1000+ patients with confirmed COVID-19 diagnosis” by Sayyed Mostafa Mostafavi, licensed under CC0 1.0. COVID19-CT-Dataset: An Open-Access Chest CT Image Repository of 1000+ patients with confirmed COVID-19 diagnosis. CT images of subjects with confirmed lung infections after positive Covid-19 diagnosis.

**Figure 13:**
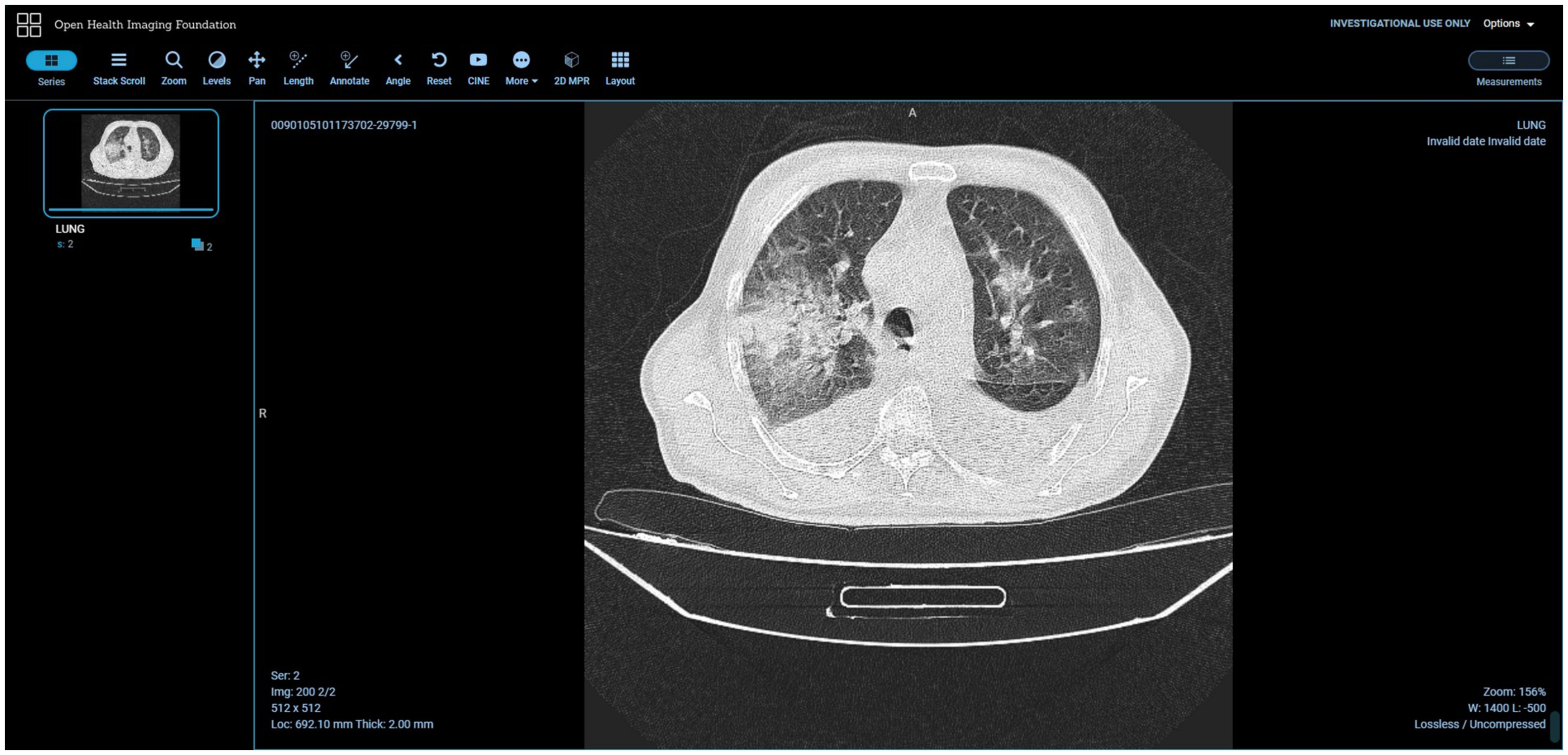
OHIF external viewer. Adapted with permission from “COVID19-CT-Dataset: An Open-Access Chest CT Image Repository of 1000+ patients with confirmed COVID-19 diagnosis” by Sayyed Mostafa Mostafavi, licensed under CC0 1.0. COVID19-CT-Dataset: An Open-Access Chest CT Image Repository of 1000+ patients with confirmed COVID-19 diagnosis. CT images of subjects with confirmed lung infections after positive Covid-19 diagnosis.

DICOM standard and its RT supplement play a special role in the research activities of some work packages of the SINFONIA project, hence the repository was conceived from the beginning for managing such files. As a result, two data management tools coexist in the repository: Girder and Orthanc. Through a DICOM plugin, Girder admits a DICOM previewer (see Figure 12) that fits in the SINFONIA project context, but an extended behavior of this plugin was implemented to expand its functionalities, such as correctly rendering DICOM files with RGB image data, support RT-DOSE, RT-PLAN and RT-STRUCT files, etc. The look-and-feel of this previewer mimics its Girder counterpart, but its internal behavior was modified to improve the user experience in response to users’ feedback. The snapshot is a PNG image (not a browser-run render anymore) and the tag list is generated after a JSON object (not using a browser-run parser for extracting tags from DICOM files). Both resources are provided by specific API endpoints of the Orthanc PACS server. This enhanced previewer also allows the display of images and videos in several usual formats (such as GIF, JPG, or PNG for images, and MP4, WebM, or Ogg for videos), and provides a listing of the hierarchy of directories and files contained in a ZIP file without the need to unzip it for inspection. See Deliverable 5.1 (23) for further details. Moreover, due to the SINFONIA partners’ needs, the external OHIF DICOM web viewer was included (Figure 13). It cannot be used for diagnosis or treatment as it is not a medical device, meaning that it is not cleared by regulatory agencies for such purposes and so it is the users’ responsibility to ensure compliance with applicable rules and regulations.

As a health data repository aimed to support massive file transfer, an SFTP server was implemented. Users can connect to the SFTP server using either GUI clients such as FileZilla or WinSCP, but user directories in the SFTP are not coupled with the Girder/Orthanc-indexed storage of the web application: the service is configured to create an isolated environment when a valid user connects to the SFTP client of their choice so that the user can only upload to, download from, and browse its folders. From this environment, a user can copy the desired files to the repository to be shared, or, equivalently, from the repository, a user can copy files to the SFTP environment to download a massive amount of data.

As sharing large datasets is a common practice, compressed files bundling them are expected to be usually uploaded to the repository. So, a useful decompressing tool was implemented featuring the chance of decompressing and saving compressed zip files in the repository. This was implemented as a brand-new model in Django, where a recursive iterator explores the .zip folder, identifies its structure, and decompresses the folder into the repository with its original subfolder hierarchy.

**Figure 14:**
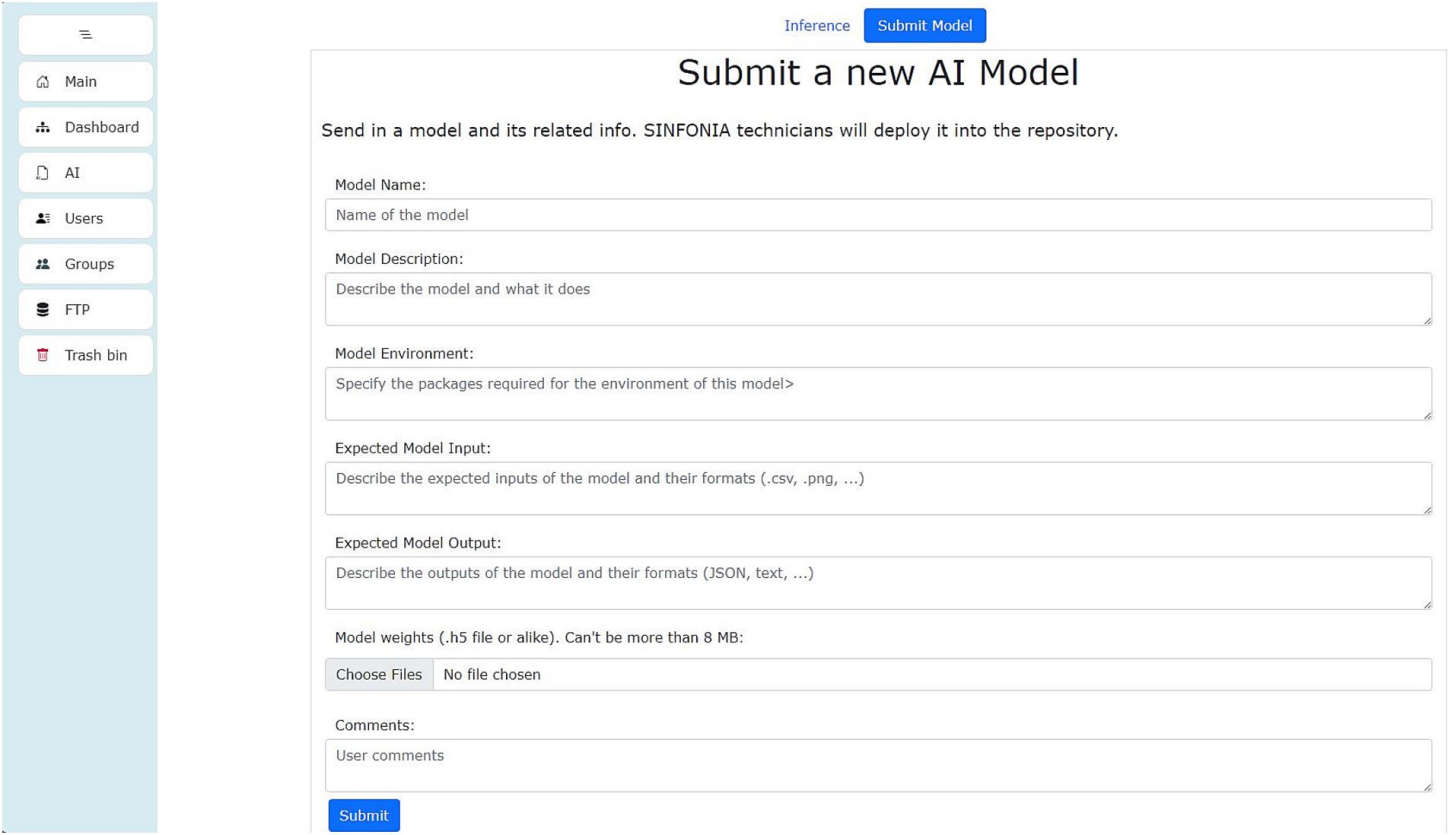
Form to submit a trained AI model.

As explained in the introduction, AI is an increasing tool in health research. One of the features of the SINFONIA repository is the possibility of sharing trained AI models. The repository itself allows the execution of said trained models with both inputs previously stored in the repository and keyboard inputs. Researchers can upload their models via the form shown in Figure 14, with the repository management team receiving and implementing them in the AI area so that they can be run by other repository users. The implementation process is not automatic, though, due to the number of frameworks an AI model can be trained with.

## 4 Discussion

The SINFONIA repository allows the project partners to share several kinds of health data files, both imaging and non-imaging, with additional viewing tools for DICOM files. Moreover, the repository is powered with an AI platform for uploading and running the AI models developed and trained by the partners within their research activities during the project.

Let us remark that the repository is motivated by specific requirements defined by its primary users, i.e., the SINFONIA project partners. It was not designed to replace or surpass pre-existing repositories or solutions backed by large development teams or years of refinement thanks to the feedback of a massive user community. Instead, it builds upon such kind of tools to create a repository tailored to the needs of the SINFONIA project. This article focuses on highlighting the successful integration of various well-established components rather than positioning the repository as a standalone software product.

Of course, although we present this integration as successful in general terms, there are functionalities of the repository that could be enhanced. For instance, regarding the management and visualization of DICOM files, there is room for improvement by implementing a finer-grained file access authorization system for the Orthanc PACS capable of replicating the permissions set by users in the repository, or by looking for alternatives to the DICOM viewers able to deal with files coming from devices with proprietary implementations of the DICOM standard or with vendor-specific information.

In turn, it can be placed among the great variety of EU-funded health imaging repositories such as Primage (58), CHAIMELEON (59), ProCΑncer-I (60), INCISIVE (61), and EuCanImage (62). All these repositories work together under the AI for health imaging (AI4HI) initiative, sharing experience and good practices toward the development of big data infrastructures based on European, ethical, and GDPR compliant, quality-controlled, cancer-related, medical imaging, and related patient data platforms, in which both large-scale data and AI algorithms will coexist. Those five projects are taking part in the European Federation for Cancer Images (EUCAIM) project (63), whose aim is to deploy a pan-European digital federated infrastructure of Findable Accessible Interoperable Reusable (FAIR) cancer-related de-identified images from Real-World.

## 5 Conclusion

This article presents the development of a cloud-based data repository tailored to the needs of the SINFONIA project. Until the end of the project in August 2024 it allowed the project partners to share several kinds of health data files, both imaging and non-imaging, with additional viewing tools for DICOM files. The repository is also powered with an AI platform for uploading and running the AI models developed and trained by the partners within their research activities during the project.

The development of the repository was driven by a hybrid software engineering methodology based on the capture of initial requirements from questionnaires circulated among project partners, which led to the release of a functional prototype after the first year of the SINFONIA project, and a continuous user validation loop that allowed us to release approximately yearly increments until the end of the project in August 2024.

At the time of this writing the repository is up and running but the access to the system and the data stored in it is restricted to SINFONIA members. The repository in its current state is compliant with applicable data protection legislation (European Union GDPR and LOPD-GDD Spanish implementation) insofar as the project plan stipulates that any data uploaded by users must have been previously de-identified and that unregistered users are not allowed access to the repository.

However, the project plan establishes as one of its final objectives to lay the foundations for the future sustainability of the repository and the data it contains. The EUCAIM initiative emerged as a great opportunity for that by integrating the SINFONIA Repository as a Federated Node. As a Federated Node, the SINFONIA repository would receive funding to adapt to the requirements of the Data Federation Framework defined by EUCAIM, which provides potential opportunities for advancing the development of the repository with improvements as the ones described in the Discussion.

## Data Availability

The original contributions presented in the study are included in the article, further inquiries on the repository source code can be directed to the corresponding author. Data collected within the SINFONIA project is out of the scope of this paper, which focuses on the SINFONIA repository as a software product.

## Nomenclature

CESGA: Galicia Supercomputing Center, Spain
EIBIR: European Institute for Biomedical Imaging Research, Austria
NCBJ: Narodowe Centrum Badań Jądrowych, Poland
OVGU: Otto-von-Guericke-Universität Magdeburg, Germany
QAELUM: QAELUM NV, Belgium
SCK-CEN: Studiecentrum Voor Kernenergie/Centre d’etude del’Energie Nucléaire, Belgium
SCO: Świętokrzyskie Centrum Onkologii, Poland
SERGAS: Servizo Galego de Saúde, Spain
SKANDION: Kommunalförbundet Avancerad Strålbehandling, Sweden
SU: Stockholms Universitet, Sweden
UGENT: Universiteit Gent, Belgium
UJK: Uniwersytet Jana Kochanowskiego W Kielcach, Poland
UNIGE: Université de Genève, Switzerland
UoC: Panepistimio Kritis/University of Crete, Greece

## References

[1] McAfee A Brynjolfsson E. Big data: the management revolution. Harv Bus Rev. (2012) 90:60–6, 68, 128. PMID: 23074865

[2] Clark K Vendt B Smith K Freymann J Kirby J Koppel P . The cancer imaging archive (TCIA): maintaining and operating a public information repository. Digit Imaging. (2013) 26:1045–57. doi: 10.1007/s10278-013-9622-7, PMID: 23884657 PMC3824915

[3] Fedorov A Longabaugh WJR Pot D Clunie DA Pieper S Aerts HJWL . NCI imaging data commons. Cancer Res. (2021) 81:4188–93. doi: 10.1158/0008-5472.CAN-21-0950, PMID: 34185678 PMC8373794

[4] Collins FS Morgan M Patrinos A. The human genome project: lessons from large-scale biology. Science. (2003) 300:286–90. doi: 10.1126/science.1084564, PMID: 12690187

[5] Benson D Lipman DJ Ostell J. Genbank. Nucleic Acids Res. (1993) 21:2963–5. doi: 10.1093/nar/21.13.2963 8332518 PMC309721

[6] Sayers EW Cavanaugh M Clark K Pruitt KD Sherry ST Yankie L . GenBank 2024 update. Nucleic Acids Res. (2024) 52:D134–7. doi: 10.1093/nar/gkad903 37889039 PMC10767886

[7] SINFONIA Project. Radiation risk appraisal for detrimental effects from medical exposure during management of patients with lymphoma or brain tumour. (2020). Available at: https://www.sinfonia-appraisal.eu (Accessed April 29, 2024).

[8] Allen C Kelly K Bollard C. Pediatric lymphomas and histiocytic disorders of childhood. Pediatr Clin N Am. (2015) 62:139–65. doi: 10.1016/j.pcl.2014.09.010, PMID: 25435117 PMC4250829

[9] Johnson KJ Cullen J Barnholtz-Sloan JS Ostrom QT Langer CE Turner MC . Childhood brain tumor epidemiology: a brain tumor epidemiology consortium review. Cancer Epidemiol Biomarkers Prev. (2014) 23:2716–36. doi: 10.1158/1055-9965.EPI-14-0207, PMID: 25192704 PMC4257885

[10] Breastcancer.org. Facts and statistics. (2024). Available at: https://www.breastcancer.org/facts-statistics (Accessed June 12, 2024).

[11] Diaz-Pinto A Alle AS Nath V Tang Y Ihsani A Asad M . MONAI label: a framework for AI-assisted interactive labeling of 3D medical images. Med Image Anal. (2024) 95:103207. doi: 10.1016/j.media.2024.103207, PMID: 38776843

[12] Koh DM Papanikolaou N Bick U Illing R Kahn CE Kalpathi-Cramer J . Artificial intelligence and machine learning in cancer imaging. Commun Med. (2022) 2:133. doi: 10.1038/s43856-022-00199-0, PMID: 36310650 PMC9613681

[13] Bi WL Hosny A Schabath MB Giger ML Birkbak NJ Mehrtash A . Artificial intelligence in cancer imaging: clinical challenges and applications. CA Cancer J Clin. (2019) 69:127–57. doi: 10.3322/caac.21552, PMID: 30720861 PMC6403009

[14] Myronakis M Stratakis J Damilakis J. Rapid estimation of patient-specific organ doses using a deep learning network. Med Phys. (2023) 50:7236–44. doi: 10.1002/mp.16356, PMID: 36918360

[15] Tzanis E Stratakis J Myronakis M Damilakis J. A fully automated machine learning-based methodology for personalized radiation dose assessment in thoracic and abdomen CT. Phys Med. (2024) 117:103195. doi: 10.1016/j.ejmp.2023.103195 38048731

[16] Tsironi F Myronakis M Stratakis J Sotiropoulou V Damilakis J. Organ dose prediction for patients undergoing radiotherapy CBCT chest examinations using artificial intelligence. Phys Med. (2024) 119:103305. doi: 10.1016/j.ejmp.2024.103305, PMID: 38320358

[17] Salimi Y Akhavanallaf A Mansouri Z Shiri I Zaidi H. Real-time, acquisition parameter-free voxel-wise patient-specific Monte Carlo dose reconstruction in whole-body CT scanning using deep neural networks. Eur Radiol. (2023) 33:9411–24. doi: 10.1007/s00330-023-09839-y, PMID: 37368113 PMC10667156

[18] Salimi Y Mansouri Z Hajianfar G Sanaat A Shiri I Zaidi H. Fully automated explainable abdominal CT contrast media phase classification using organ segmentation and machine learning. Med Phys. (2024) 51:4095–104. doi: 10.1002/mp.17076, PMID: 38629779

[19] Salimi Y Shiri I Akavanallaf A Mansouri Z Arabi H Zaidi H. Fully automated accurate patient positioning in computed tomography using anterior–posterior localizer images and a deep neural network: a dual-center study. Eur Radiol. (2023) 33:3243–52. doi: 10.1007/s00330-023-09424-3, PMID: 36703015 PMC9879741

[20] Salimi Y Shiri I Akhavanallaf A Mansouri Z Sanaat A Pakbin M . Deep learning-based calculation of patient size and attenuation surrogates from localizer image: toward personalized chest CT protocol optimization. Eur J Radiol. (2022) 157:110602. doi: 10.1016/j.ejrad.2022.110602, PMID: 36410091

[21] Salimi Y Shiri I Akhavanallaf A Mansouri Z Saberi Manesh A Sanaat A . Deep learning-based fully automated Z-axis coverage range definition from scout scans to eliminate overscanning in chest CT imaging. Insights Imaging. (2021) 12:162. doi: 10.1186/s13244-021-01105-3, PMID: 34743251 PMC8572075

[22] European Union. General data protection regulation. Strasbourg. Available at: https://eur-lex.europa.eu/eli/reg/2016/679/oj (Accessed April 29, 2024).

[23] SINFONIA Project. Deliverable 5.1. Santiago de Compostela; (2020). Available at: https://www.sinfonia-appraisal.eu/wp-content/uploads/2022/10/SINFONIA_Deliverable_D5.1_final.pdf (Accessed April 29, 2024).

[24] SINFONIA Project. Deliverable 5.2. Santiago de Compostela; (2020). Available at: https://www.sinfonia-appraisal.eu/wp-content/uploads/2024/01/D5.2-Platform-description-data-model-and-protocols.pdf (Accessed April 29, 2024).

[25] SINFONIA Project. Deliverable 5.3. (2020). Available at: https://www.sinfonia-appraisal.eu/sinfonia-research-results/ (Accessed April 29, 2024).

[26] Crinnion J. Evolutionary systems development: a practical guide to the use of prototyping within a structured systems methodology. New York: Springer (1992).

[27] Pressman RS. Software engineering: a practitioner’s approach. 7th ed. New York: McGraw Hill Education (2010).

[28] DICOM Standars Commitee. Digital Imaging and communication in medicine (DICOM). Available at: https://www.dicomstandard.org/ (Accessed April 29, 2024).

[29] Aylward SR. On line collection of medical images repositories. (2024). Available at: https://www.aylward.org/notes/open-access-medical-image-repositories (Accessed June 12, 2024).

[30] Fisher R. CVonline: Image Databases. (2016). Available at: https://homepages.inf.ed.ac.uk/rbf/CVonline/Imagedbase.htm#biomed (Accessed June 12, 2024).

[31] The National Institutes of Health (NIH) a part of the U.S. Department of Health and Human. NeuroImaging Tools and Resources Collaboratory (NITRC). Available at: https://www.nitrc.org/ (Accessed June 12, 2024).

[32] Kitware, Inc. Girder, a data management platform. (2014–2018). Available at: https://girder.readthedocs.io/ (Accessed April 29, 2024).

[33] Software Engineering Institute. Software architecture. (2024). Available at: https://www.sei.cmu.edu/our-work/software-architecture/ (Accessed May 6, 2024).

[34] Eckerson WW. Three tier client/server architectures: achieving scalability, performance, and efficiency in client server applications. Open Inf Syst. (1995) 3:46–50.

[35] PostDICOM. What is PACS? And how does it work? (2024). Available at: https://www.postdicom.com/en/blog/what-is-pacs-and-how-does-it-work (Accessed May 5, 2024).

[36] Dejwakh V. The model-view-controller (MVC) pattern. (2020). Available at: https://vahid.blog/post/2021-04-16-understanding-the-model-view-controller-mvc-pattern/ (Accessed April 29, 2024).

[37] Choplin RHE Boehme JM Maynard CD. Picture archiving and communication systems: an overview. Radiographics. (1992) 12:127–9. doi: 10.1148/radiographics.12.1.1734458 1734458

[38] Gillis AS. Secure File Transfer Protocol (SSH File Transfer Protocol). Available at: https://www.techtarget.com/searchcontentmanagement/definition/Secure-File-Transfer-Protocol-SSH-File-Transfer-Protocol (Accessed April 29, 2024).

[39] Network Working Group. Lightweight Directory Access Protocol (LDAP): The Protocol. Available at: https://www.rfc-editor.org/rfc/rfc4511 (Accessed May 2, 2024).

[40] OpenLDAP Foundation. Introduction to OpenLDAP Directory Services. (2011–2021). Available at: https://www.openldap.org/doc/admin24/intro.html (Accessed April 29, 2024).

[41] Docker Inc. Docker. (2024). Available at: https://www.docker.com/ (Accessed April 29, 2024).

[42] Mongo DB Inc. Introduction to Mongo DB. (2024). Available at: https://www.mongodb.com/docs/manual/introduction/ (Accessed April 29, 2024).

[43] UCLouvain. Orthanc. (2021–2023). Available at: https://www.orthanc-server.com/static.php?page=about (Accessed April 29, 2024).

[44] Digital Imaging and Communication in Medicine. DICOMweb. Available at: https://www.dicomstandard.org/using/dicomweb (Accessed April 29, 2024).

[45] Django Software Foundation. Overview. Django Project. (2005–2024). Available at: https://www.djangoproject.com/start/overview/ (Accessed April 29, 2024).

[46] Holovaty A Kaplan-Moss J. The Django Book. (2022). Available at: https://readthedocs.org/projects/djangobook/downloads/pdf/latest/ (Accessed April 29, 2024).

[47] Ziegler E Urban T Brown D Petts J Pieper SD Lewis R . Open health imaging foundation viewer: an extensible open-source framework for building web-based imaging applications to support cancer research. JCO Clin Cancer Inform. (2020) 4:336–45. doi: 10.1200/CCI.19.00131, PMID: 32324447 PMC7259879

[48] OnlyOffice. OnlyOffice Docs. (2009–2024). Available at: https://www.onlyoffice.com/en/office-suite.aspx (Accessed April 29, 2024).

[49] The Mathworks Inc. Matlab Runtime. (2022). Available at: https://mathworks.com/products/compiler/matlab-runtime.html (Accessed May 20, 2024).

[50] MongoDB. BSON (Binary JSON) Serialization. Available at: https://bsonspec.org/ (Accessed April 29, 2024).

[51] The PostgreSQL Global Development Group. About PostgreSQL. (1996–2024). Available at: https://www.postgresql.org/about/ (Accessed April 29, 2024).

[52] Osixia GB. OpenLDAP. (2024). Available at: https://github.com/osixia/docker-openldap (Accessed April 29, 2024).

[53] F5 Inc. About NGINX. Available at: https://www.nginx.com/about/ (Accessed April 29, 2024).

[54] The Apache Software Foundation. Apache HTTP server. (1997–2024). Available at: https://httpd.apache.org/ (Accessed May 5, 2024).

[55] Spanish Lex. Ley Orgánica 3/2018, de 5 de diciembre, de Protección de Datos Personales y garantía de los derechos digitales. Boletín Oficial del Estado. (2018) 294:119788–857.

[56] SINFONIA Project. SINFONIA Anonymization Guidelines. Available at: https://sinfonia.cesga.es/SINFONIA-Anonymization-Guidelines.pdf (Accessed May 5, 2024).

[57] European Commission. Proposal for a REGULATION OF THE EUROPEAN PARLIAMENT AND OF THE COUNCIL on THE European health data space. (2022). Available at: https://eur-lex.europa.eu/legal-content/EN/TXT/?uri=celex%3A52022PC0197 (Accessed May 2, 2024).

[58] Martí-Bonmatí L Alberich-Bayarri Á Ladenstein R Blanquer I Segrelles JD Cerdá-Alberich L . PRIMAGE project: predictive in silico multiscale analytics to support childhood cancer personalised evaluation empowered by imaging biomarkers. Eur Radiol Exp. (2020) 4:22. doi: 10.1186/s41747-020-00150-9 32246291 PMC7125275

[59] Bonmatí LM Miguel A Suárez A Aznar M Beregi J Fournier L . CHAIMELEON project: creation of a pan-European repository of health imaging data for the development of AI-powered cancer management tools. Front Oncol. (2022) 12:742701. doi: 10.3389/fonc.2022.742701, PMID: 35280732 PMC8913333

[60] ProCAncer-I Project. An AI Platform integrating imaging data and models, supporting precision care through prostate cancer’s continuum. Malta. Available at: ttps://www.procancer-i.eu/ (Accessed April 29, 2024).

[61] Lazic I Agullo F Ausso S Alves B Barelle C Berral JL . He holistic perspective of the INCISIVE project—artifcial intelligence inscreening mammography. Appl Sci (Basel). (2022) 12:8755. doi: 10.3390/app12178755

[62] EuCanImage Project. An European cancer imaging platform for enhanced Artificial Intelligence in oncology. (2020). Available at: https://eucanimage.eu/ (Accessed April 29, 2024).

[63] EUCAIM project. European Cancer Imaging Initiative. (2024). Available at: https://cancerimage.eu/ (Accessed May 2, 2024).

